# A pilot randomized controlled study of a digital art therapy paradigm for children with ASD: integrating pivotal response treatment into video animation and physical model design

**DOI:** 10.3389/fpsyt.2026.1894877

**Published:** 2026-08-26

**Authors:** Huili She, Xinyi Dai, Ting Deng, Ruhao Liu, Wenting Jiang, Xufeng Ma

**Affiliations:** 1 School of Art and Design, Anhui University of Technology, Ma’anshan, Anhui, China; 2 School of Journalism and New Media, Xi’an Jiaotong University, Xi’an, Shaanxi, China; 3 College of Arts & Media, Tongji University, Shanghai, China

**Keywords:** autism spectrum disorder, cross-media design, digital art therapy, pivotal response treatment, social functioning

## Abstract

**Background:**

The integration of digital technology into art therapy for children with autism spectrum disorder (ASD) represents an emerging trend, yet systematic design frameworks that translate evidence-based interventions into digital media remain underdeveloped. This study developed and evaluated a digital art therapy paradigm that structurally integrates pivotal response treatment (PRT) into video animation design while maintaining cross-media consistency with physical generalization tools.

**Methods:**

Based on the behavioral characteristics of children with ASD, 12 sets of video animations integrating PRT and corresponding physical scene models were custom developed. Thirty-eight children with mild to moderate ASD were randomly assigned to an intervention group (PRT-based video animation with physical models, n=19) and a control group (conventional video animation, n=19) for a 12-week intervention. Pre- and postintervention assessments were conducted using four dimensions of the Autism Treatment Evaluation Checklist (ATEC) and behavioral observation.

**Results:**

Compared with the control group, the intervention group demonstrated significantly greater improvements across all ATEC dimensions (language communication: F(1,36) = 67.74, p < 0.001, ηp² = 0.755; social skills, F(1,36) = 80.69, p < 0.001, ηp² = 0.786; sensory/cognitive awareness: F(1,36) = 55.69, p < 0.001, ηp² = 0.717; health/physical behavior, F(1,36) = 36.94, p < 0.001, ηp² = 0.627. The percentage of children who reported “ no observable behavioral response” in the intervention group decreased from 35% to 10%.

**Conclusions:**

This pilot randomized controlled study provides preliminary evidence for the feasibility and efficacy of a multi-component, PRT-informed digital and physical intervention package for children with ASD. The intervention is characterized by three design principles: (1) theoretical translation-–embedding the structured steps of PRT into digital interaction logic; (2) cross-media consistency-–maintaining visual and functional coherence between video animations and physical generalization tools; and (3) progressive scaffolding--sequencing intervention content from foundational cognition to complex social skills. These findings offer a preliminary design framework for the development of digital art therapy interventions for children with ASD. Future large-checklist replication studies are needed to further validate this approach.

## Highlights

- In this study, a digital art therapy model based on pivotal response treatment (PRT) was constructed to organically integrate intervention methods with digital technology to form a structured therapeutic pathway. The results demonstrated a significant improvement in social functioning in children with ASD.
- The PRT-integrated video animation intervention program used by the intervention group resulted in significant improvements in the four core dimensions of verbal communication, social skills, sensory/cognitive awareness, and health/physical behavior, with the improvement effects being significantly better than those in the conventional video intervention group.
- The combination of visual, situational digital media with generalization training led to significantly increased behavioral activity levels for the children in the intervention group. The percentage of children who reported “ no observable behavioral response” decreased from 35% before the intervention to 10%, indicating that this paradigm can effectively stimulate participation motivation and behavioral expression in children.
- This research confirmed the feasibility of integrating the structured steps of PRT with digital art media. An integrated intervention design framework of “video animation + physical models + generalization tools” was proposed, which provided replicable and scalable practical references for using digital art therapy for children with ASD.

## 1 Introduction

Autism spectrum disorder (ASD) is a neurodevelopmental disorder characterized by social communication impairments and restricted, repetitive patterns of behavior (1, 2). Currently, specific pharmacological treatments with significant efficacy for the core symptoms of ASD are lacking (3). Therefore, rehabilitation training primarily based on educational interventions remains important for improving the core functions and enhancing the social adaptability of children with ASD (4, 5). Art therapy (such as music, visual arts, etc.), as a nonpharmacological intervention, has shown promise in promoting emotional expression, social interaction, and behavioral regulation. It has been widely applied in rehabilitation practices for children with ASD (6–9).

Social functioning is the core ability children with ASD need to help them integrate into society. It spans multiple developmental dimensions, such as cognition, language, emotion, and behavior, and has a decisive effect on their lifelong development (10). Specifically, social functioning encompasses the ability to understand social rules, recognize others’ emotions, engage in effective interpersonal interactions, adapt to diverse environments, and maintain emotional and behavioral stability (11–13). Deficits in this function not only constrain the language and social development of children with ASD, affecting their mental health and quality of life but also impose significant long-term burdens on the families of these individuals and society (14, 15).To further clarify the operational definition of “digital art therapy” in this study, we define it as a structured, PRT-informed intervention paradigm that leverages digital animation as the primary cognitive acquisition medium and physical art-based generalization activities as the transfer and embodiment platform. Specifically, this paradigm operates through two integrated phases:

Phase 1: Digital Cognitive Acquisition. Children acquire foundational cognitive and operational knowledge (e.g., social rules, procedural steps) through interaction with rich colors, dynamic characters, and interactive buttons within the digital interface. This phase embeds PRT principles (motivation, choice, task decomposition, immediate feedback) into the digital interaction logic.

Phase 2: Embodied Generalization and Transfer. Children seamlessly transition from segmented digital learning to theme-related generalization training, which includes but is not limited to: artistic creation (e.g., drawing, clay modeling), functional operational training (e.g., categorization games, shaping), and social role-playing (e.g., shopping simulations, cooperative tasks). The physical scene models are 1:1 replications of the digital environments, maintaining visual and functional consistency to facilitate skill transfer.

The ultimate goal of this two-phase design is to concretize abstract social rules and procedural steps into tangible, embodied actions, thereby enabling an effective transfer from “digital cognition” to “real-world competence.”

## 2 Literature review

Various evidence-based practice methods have been developed for intervening in ASD. Among them, applied behavior analysis (ABA) (16), cognitive behavioral therapy (CBT) (17), early intensive behavioral intervention (EIBI) (18), and pivotal response treatment (PRT) (19) are currently the main, widely applied intervention methods (2). PRT originates from applied behavior analysis theory (20). The core of this intervention focuses on “pivotal” areas such as motivation, self-management, self-initiation, and responsiveness to multiple cues (21). It aims to promote widespread functional improvements in social, communication, and behavioral domains by enhancing these core capabilities (22, 23).

As a structured and individualized naturalistic intervention strategy, PRT particularly emphasizes promoting the development of children’s social functioning within daily life and interactive contexts to alleviate the core symptoms of ASD (24, 25). Early evidence regarding the effectiveness of PRT came primarily from small-sample studies (26). In recent years, randomized controlled trials (RCTs) have gradually become the primary research method for validating the effects of such interventions (27, 28). For example, a randomized controlled trial by de Korte et al. revealed that children with ASD who received a PRT intervention performed significantly better in terms of demonstrating social interaction skills than those in the conventional intervention group did (22). Furthermore, research has shown that PRT helps improve the quality of parent–child interactions and reduces parenting stress (29). These findings provide empirical support for the application of PRT in promoting the development of social functioning in children with ASD and providing family support.

Research has indicated that approximately 74% of children with ASD have received at least one form of complementary and alternative therapy (30, 31), with art therapy forms such as music, dance, and drama being particularly common. With the development of digital technology, art therapy based on digital media has shown potential for enhancing multidomain skills in children with ASD (32). Compared with traditional interventions, digital methods can strengthen core instructional stimuli and reduce environmental distractions, thereby alleviating issues of over selective attention in children with ASD (33) and promoting the development of their social communication and interactive behaviors (34, 35). Common forms of digital interventions include video demonstrations (36, 37) and interactive products (38, 39). Their application value in resource-limited settings is becoming increasingly recognized (40), garnering widespread attention from parents and academics. Al Jaffal’s (2023) systematic review further confirmed that video−based interventions (VBIs) have positive intervention effects on vocational skills, daily living skills, and academic skills for adolescents and young adults with ASD. Moreover, the intervention effects were found to be maintained and generalized, and most studies also reported that VBIs have high social validity.

Despite promising developments in both PRT and digital interventions, a critical gap remains in terms of how to systematically translate the therapeutic logic of PRT into concrete design specifications for digital art therapy products. Current research tends to treat intervention methods and digital media design as separate disciplinary domains, with insufficient attention given to the “translation mechanism” between theory and design. Furthermore, while digital interventions offer advantages in terms of standardization and accessibility, concerns persist regarding skill generalization from screen-based environments to real-life contexts. This study addresses these gaps by proposing an integrated design framework that embeds PRT principles into both digital (video animation) and physical (scene models, operational tools) media while maintaining cross-media consistency to facilitate skill transfer.

This study focuses on the video animation form under the intervention method. It aims to systematically enhance the social skills and behavioral functioning of adolescents with ASD by developing digital therapeutic materials suitable for ASD adolescents that integrate digital media production tools with animation production technology. Although scholars have explored the use of digital media for art therapy (41, 42) and related methods (43, 44), the current research still has obvious limitations. Intervention training methods and digital media design typically belong to different disciplinary fields. Methods for deeply integrating the two fields and their actual effects still lack sufficient empirical evidence. Therefore, this study attempts to construct an integrated digital art therapy model that combines an evidence-based intervention method—pivotal response treatment (PRT)—with digital art media. The effects compared with those of general digital interventions are then examined through controlled experiments. The specific research questions are as follows:

1. Theoretical Translation: How can the core components of PRT (motivation, choice, task analysis, child initiation, feedback, generalization, and reinforcement) be systematically translated into design specifications for digital art therapy media?
2. Design Factors: What specific design elements—at the levels of visual presentation, interaction logic, narrative structure, and cross-media consistency—contribute to the enhancement of social functioning in children with ASD?
3. Generalization Mechanism: How does the deliberate alignment between digital scenarios and physical models facilitate skill transfer, and what are the observable behavioral manifestations of this transfer during an intervention?

**Figure 1:**
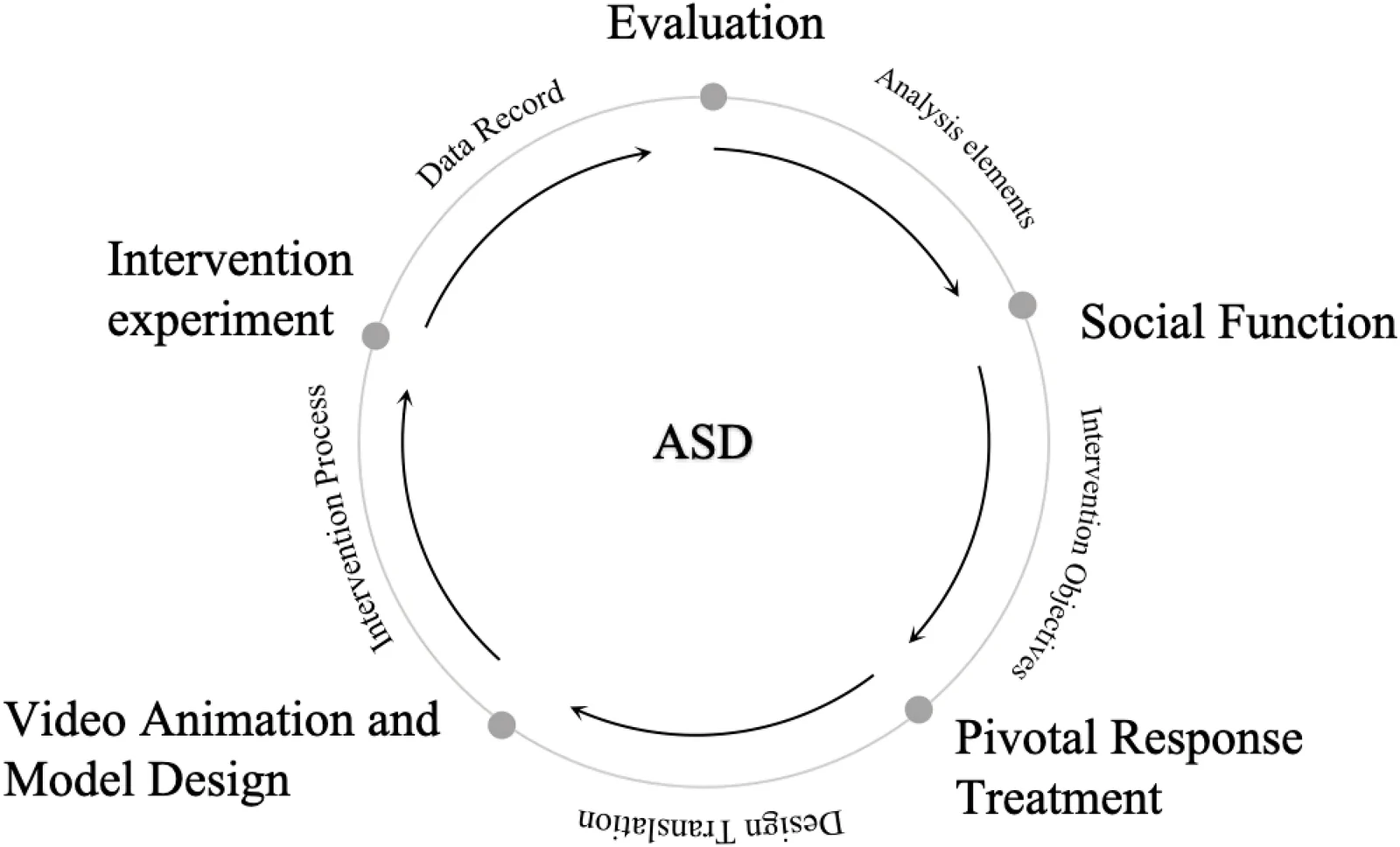
Theoretical framework.

The literature review reveals two critical gaps: first, the theoretical logic of PRT has not been systematically translated into concrete design specifications for digital art therapy; second, existing digital interventions still face a transfer barrier from screen-based learning to real-world application in terms of skill generalization. To address these gaps, this study proposes a “PRT–Design–Mechanism” theoretical framework. Anchored in the seven core steps of PRT (motivation elicitation, choice provision, task decomposition, child initiation, immediate feedback, generalization training, and reinforcement), this framework translates the intervention logic into interactive specifications for video animations and generalization pathways through physical models, guided by three design principles: visual consistency, progressive task scaffolding, and multimodal reinforcement. The framework ultimately targets the enhancement of social functioning, providing a theoretical basis for subsequent intervention development and empirical validation (see **Figure 1**).

## 3 Methods

### 3.1 Research subjects and environment

#### 3.1.1 Ethical considerations

This study was approved by the Ethics Committee of Anhui University of Technology (Approval No.: YXLLSP20241003). The study was conducted in full compliance with the Declaration of Helsinki. All participating children’s legal guardians provided written informed consent before enrollment (see **Supplementary Material** for the full consent form). Guardians were fully informed of the study purpose, intervention procedures, potential risks, data confidentiality measures, and the right to withdraw at any point without penalty. Given that this intervention is non-pharmacological and primarily behavior-based, all intervention activities were conducted in autism-specific classrooms within a special education school, under the joint guidance of autism specialist teams from both the special education school and a local hospital. Therefore, this study was not registered in a clinical trial registry prior to its initiation—we acknowledge this limitation and are committed to improving the transparency of future.The study involved minimal risk to participants, and all procedures were designed to ensure the safety and comfort of the children throughout the intervention.

#### 3.1.2 Participants and study design

All participating children received systematic diagnostic assessments at the Department of Child Health/Developmental Behavior, Ma’anshan Maternal and Child Health Hospital prior to school enrollment. ASD diagnosis followed DSM-5 criteria, and the school assigned participants to appropriate classes and grade levels according to their severity and cognitive functioning.

Between September 2023 and June 2025, the team recruited 38 children aged 7–12 with mild-to-moderate ASD from local special education schools, with 19 assigned to the intervention group (16 male, 3 female; mean age = 8.8 years, SD = 1.9, range: 6.4–12.2) and 19 to the control group (15 male, 4 female; mean age = 9.4 years, SD = 1.8, range: 6.8–12.5). Drawing on the medical and behavioral characteristics of this population and grounded in the PRT framework, we developed 12 sets of PRT-integrated video animations, along with corresponding physical models and props for generalization training.Baseline characteristics of the participants are presented in **Table 1**. The intervention and control groups were comparable at baseline across all demographic and clinical measures. Independent-samples t-tests revealed no significant differences between groups in age (t(36) = -0.98, p = 0.334) or baseline ATEC subchecklist scores (Language: p = 0.219; Social: p = 0.807; Sensory: p = 0.628; Behavior: p = 0.298). Chi-square tests indicated no significant differences in sex distribution (χ²(1) = 0.11, p = 0.740) or CARS severity classification (all participants scored within the 30–36 mild-to-moderate range). All participants were assessed using the Wechsler Intelligence Scale for Children (age-appropriate version) and a standardized language development test prior to enrollment; the school employed a dual-classification system based on these assessments to assign participants to appropriate grade-level cognitive classes, ensuring comparable cognitive and linguistic profiles across groups. These results confirm successful randomization and baseline equivalence between the two groups (see **Table 2**).

**Table 1:** Intervention group video animation courses and intervention targets.

No.	Name	Theme	Category	Activity
1	Kitchen Chapter – Making Noodles	manipulative ability	Cognitive	Tear the noodles, then color them.
2	Kitchen Section-Tableware Usage	manipulative ability	Cognitive	Picking beans
3	Kitchen Chapter-Kitchenware Cognition	Security Awareness	Cognitive	Ultra-light clay (kitchenware)
4	Kitchen Chapter-Sour, Sweet, Bitter, Spicy, and Salty	Taste experience	Cognitive	Taste experience
5	Kitchen Chapter – Making Dumplings	Farm Experience	Social	Dumpling-Making Experience (Super Light Clay)
6	Life Chapter – Item Classification	Life Management	Practical	Storage Experience (Category)
7	Life Chapter-Button and Zipper	Self-care Skills	Practical	Zipper, button
8	Supermarket Section – Shopping at the Supermarket	Buy items	Social	Supermarket Experience (Shopping List)
9	Supermarket Chapter-Understanding RMB	Use of money	Social	Supermarket shopping experience (coins)
10	Farm Chapter-Plant a Small Seed	Growth Experience	Practical	Planting Experience
11	Farm Chapter: Animal Friends	Animal cognition	Cognitive	Ultra-light clay (cow)
12	Good morning, eggs!	Daily life,	Cognitive	Fried eggs (delicious fried eggs)

**Table 2:** Baseline characteristics of participants.

Characteristic	Intervention group (n=19)	Control group (n=19)	Test statistic	p-value
Age (years), M (SD)	8.8(1.9)	9.4(1.8)	t(36)=-0.98	0.334
Sex (Male/Female)	16/3	15/4	χ²(1) = 0.11	0.740
ATEC Language (M, SD)	22.33(1.30)	23.00(1.28)	F(1,36) = 1.60	0.219
ATEC Social (M, SD)	27.25(0.87)	27.33(0.78)	F(1,36) = 0.06	0.807
ATEC Sensory (M, SD)	27.75(0.62)	27.58(1.00)	F(1,36) = 0.24	0.628
ATEC Behavior (M, SD)	8.50(0.52)	8.75(0.62)	F(1,36) = 1.14	0.298

ATEC, Autism Treatment Evaluation Checklist. All participants were diagnosed with mild-to-moderate ASD based on DSM-5 criteria and CARS assessment (scores 30–36). Cognitive and language assessments were administered at the Department of Child Health/Developmental Behavior, Ma’anshan Maternal and Child Health Hospital prior to school enrollment.

Following enrollment, participants were randomly assigned in a 1:1 ratio using a computer-generated random sequence concealed in sealed, opaque, sequentially numbered envelopes. After baseline assessment and confirmation of eligibility, the envelopes were opened by an independent researcher, who notified the therapists of the group assignments. Due to the visible differences in intervention materials, therapists and children could not be blinded to group allocation. However, the two ATEC outcome assessors were blinded to group assignment and took no part in intervention delivery. Behavioral coders were also blinded to both group and time point, as all video recordings were coded using anonymized filenames containing only participant IDs and time identifiers. Data analysis was conducted by a statistician blinded to coding and group status.

**Figure 2:**
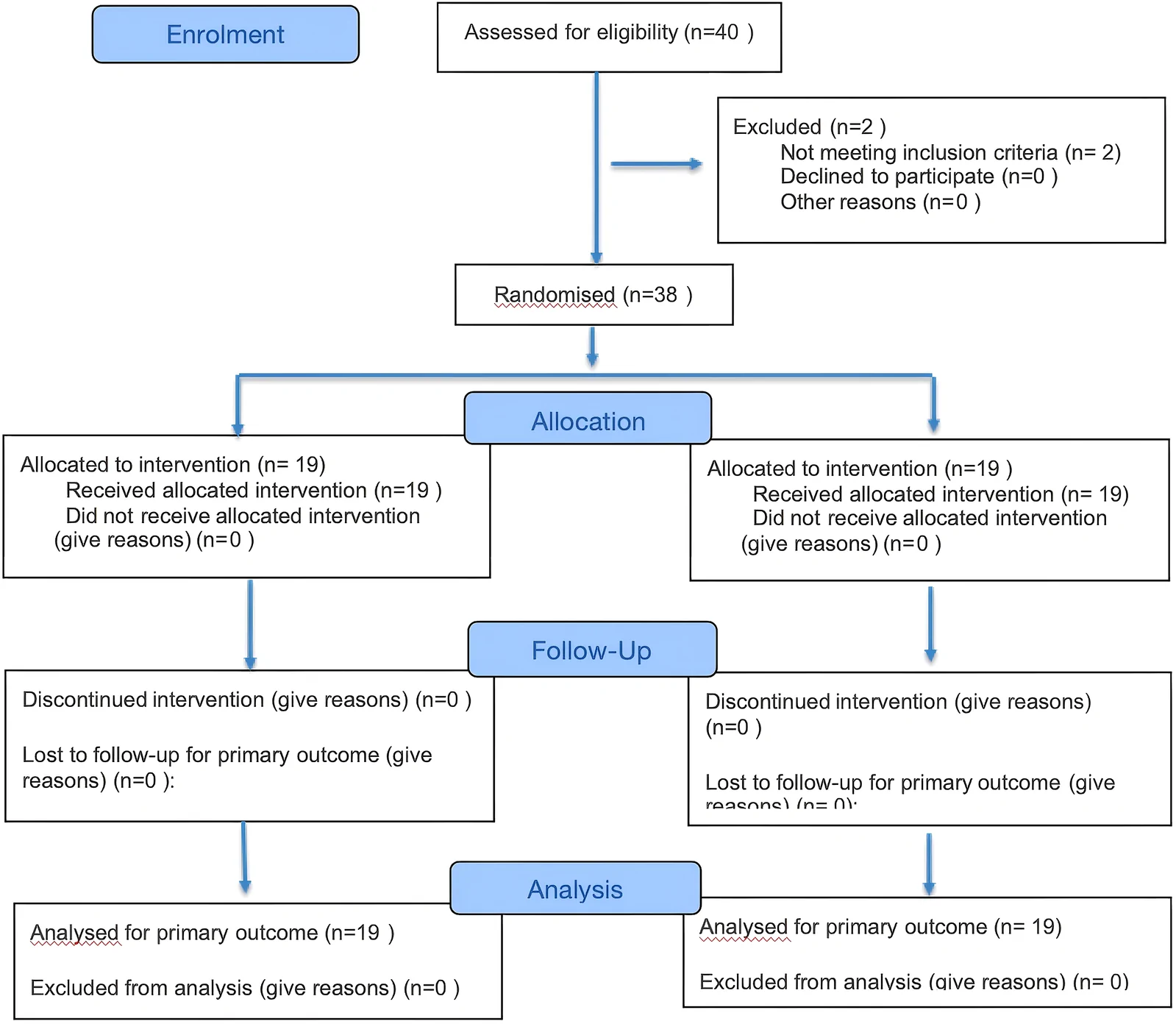
CONSORT flowchart.

All pre-test assessments, interventions, and generalization training were carried out in standard autism education classrooms equipped only with materials necessary for the research. The entire intervention was delivered by trained therapists from the school’s autism education department. The participant recruitment, randomisation, allocation, follow-up, and analysis procedures are summarised in the CONSORT flow diagram (**Figure 2**).

### 3.2 Materials

Materials included video cameras, teaching whiteboards, tangible reinforcers, recording tools, and assessment tools to determine if participants met the research criteria. These included:

- Diagnostic and Statistical Manual of Mental Disorders, Fifth Edition (DSM-5) (45).
- Parent and Teacher Informed Consent Forms.
- Autism Treatment Evaluation Checklist (ATEC).
- Behavioral checklist (self-developed).

#### 3.2.1 Autism treatment evaluation checklist (ATEC)

The Autism Treatment Evaluation Checklist (ATEC), developed by the Autism Research Institute, is a standardized, freely accessible tool for assessing symptom severity and intervention effects in individuals with ASD aged 2–18. It is widely used in clinical and academic research due to its strong reliability and validity (46).

This study used the Chinese version of the ATEC. ATEC assessments were completed by the same two independent graduate student assessors at both pre- and post-intervention time points. These assessors specialized in autism research, were not the classroom teachers, did not participate in any intervention delivery, and had no daily contact with the participating children. Assessment integrated three sources of information: (1) daily behavioral records from classroom therapists; (2) structured parent interviews regarding children’s home performance; and (3) coded observations of standardized pre- and post-test video clips. Final scores were independently determined by the two assessors based on these sources. To ensure blinding: (1) assessors had no access to the allocation sequence or intervention delivery records; (2) all pre- and post-test videos were stripped of temporal labels and randomly coded as ‘T1/T2’ prior to assessment, and assessment was conducted through video coding of standardized recordings; (3) since the thematic content of videos was identical across both groups (e.g., both featured “Kitchen—Making Noodles”), parents only knew that their child participated in video-animation activities and could not distinguish which group their child belonged to; therefore, there was no risk of parents inadvertently revealing group assignment information to assessors; (4) assessors were unaware of the study’s directional hypotheses and made final scoring decisions independently. After coding completion, data were handed to an independent statistician for unblinded analysis research.

Scores for the four ATEC dimensions (speech/language, sociability, sensory/cognitive awareness, and health/physical behavior) were recorded.

“The Chinese version of the ATEC has been validated in prior studies conducted in Chinese populations (46), demonstrating good internal consistency (Cronbach’s α > 0.80) and test-retest reliability.

#### 3.2.2 Behavioral checklist

A self-developed behavioral observation checklist was used to record children’s classroom behaviors. The checklist items were derived from the target behavioral domains of PRT (motivation, social initiation, and responsivity to multiple cues) and classroom engagement indicators. Three specific behaviors were coded: raising one’s hand to speak (as a measure of verbal initiation), social interaction (e.g., peer-directed eye contact, gesturing, or verbal exchange), and behavioral performance (e.g., compliance with classroom rules, absence of disruptive behaviors). Each session was divided into eight 5−minute intervals, and coders recorded the presence or absence of each behavior per interval. Content validity was established through review and revision by three special education experts and two senior PRT therapists. Inter−rater reliability was calculated using Cohen’s Kappa coefficient; two independent coders coded 30% of randomly selected video recordings, yielding an average Kappa of 0.89 (range: 0.82–0.94), indicating high inter−rater agreement. The percentage of children exhibiting a given behavior was calculated as (number of children who exhibited the behavior at least once during the observation period ÷ total number of children in that group) × 100%.

### 3.3 Design principles for PRT-integrated digital art therapy

The design of the intervention materials for the intervention group was guided by four principles derived from the PRT theoretical framework and the perceptual-cognitive characteristics of children with ASD:

1. Motivational Scaffolding through Visual Affordance. On the basis of PRT’s emphasis on child interest and choice, the video animation interface was designed with multiple selectable task options, allowing children to initiate interactions according to their preferences. Visual cues (e.g., highlighted buttons, character expressions) were strategically employed to signal action opportunities without relying on verbal instructions.
2. Progressive Task Decomposition. In alignment with the PRT task analysis component, each thematic course was divided into sequential subtasks of increasing complexity. Early sessions targeted foundational cognitive skills (e.g., object recognition), whereas later sessions focused on social coordination and problem-solving.
3. Cross-Media Visual Consistency. To reduce the cognitive load and facilitate generalization, strict visual consistency between all the video animations and corresponding physical models was maintained. Scene layouts, object placement, color schemes, and character designs were replicated at a 1:1 scale between the digital interface and the physical training environment. This principle addresses the documented challenge of skill transfer in ASD interventions by minimizing contextual discontinuity.
4. Immediate and Natural Reinforcement. Feedback mechanisms were embedded at multiple levels: visual prompts within the animation (e.g., character nodding), verbal encouragement from the therapist during generalization training, and tangible rewards (e.g., preferred toys) upon task completion. This multilayered reinforcement aligns with the natural reinforcement principle of PRT while accommodating the sensory preferences of children with ASD.

### 3.4 Independent variable

The independent variable in this study was the intervention method, which was divided into two groups:

- Intervention Group: The team designed 12 sets of video animations, including accompanying physical models and corresponding generalization training, based on the PRT method.
- Control Group: A conventional video animation intervention was employed that included themes consistent with those of the intervention group, including corresponding generalization training.

The difference between the intervention and control groups lay in four aspects: (1) Video format: the intervention group received interactive PRT-embedded animations, whereas the control group viewed linear animations of the same theme and duration without interactive components; (2) Materials: the intervention group used 1:1 physical scene models for generalization training, while the control group had no access to these models and only received unstructured art materials after video viewing; (3) Instructional strategy: the intervention group incorporated tiered prompts, verbal guidance, and immediate audiovisual feedback within the PRT framework, whereas the control group received none of these; (4) Reinforcement: the intervention group received immediate task−contingent reinforcement (e.g., praise, tokens), while the control group only received a fixed participatory gift at session end. Both groups were matched on dose (weekly, 45 min, 12 weeks) and therapist contact time (see **Table 3**).

**Table 3:** Comparison of intervention factors between the experimental and control groups.

Dimension	Intervention group	Control group
Dose	45 min/session, once weekly, for 12 weeks	
Therapist Contact	Delivered by trained therapists with consistent presence and guidance throughout the session	
Reinforcement	Immediate task-contingent reinforcement embedded at multiple levels	Only a fixed participatory gift provided at the very end of each session
Interactivity	Interactive video animations with clickable buttons, scenario selection, and drag-and-drop operations	Linear video animations played in a fixed sequence without any interactive operation nodes
Physical Materials	1:1 scale physical scene models and corresponding operational props provided for generalization training	only unstructured art materials
Generalization Activities	Structured generalization training with clear skill-transfer goals, requiring children to apply video-learned steps to physical models	Unstructured art activities consistent with the theme (e.g., free coloring, sticker play) without strict skill correspondence to the video content
Prompts	Tiered prompts (partial prompting → independent completion)	No content-related prompts provided
Feedback	Instant Feedback	No feedback
Task Structure	Progressive task decomposition, with clear sub-goals for each module	No systematic difficulty progression or strict skill correspondence across sessions

### 3.5 Dependent variable

The dependent variable in this study was social functioning, which was measured through the following dimensions and indicators:

- Core Dimensions: Assessed using the ATEC, which includes 77 items in total within the following four categories: language communication, social interaction, sensory perception, and health behaviors. The first three categories employ a 3-point Likert scale ranging from 0 to 2, whereas the latter category uses a 4-point Likert scale ranging from 0 to 3. Higher scores indicate more severe autism symptoms (47). Consequently, the ‘pre- and postintervention ATEC total scores and subchecklist scores’ in this study serve as the primary basis for evaluating the intervention program’s efficacy.
- Supplementary Validation Indicators: Behavioral observation data included the frequency of verbal initiations, degree of participation in social interactions, and proportions of various behaviors. Social validity involved the collection of satisfaction evaluations of the intervention effects from parents and therapists through questionnaires and interviews.

### 3.6 Research design

In this study, PRT was used as the theoretical framework, and video animation was used as the digital art therapy medium. First, on the basis of the PRT theoretical model, an intervention model and design model suitable for art therapy for children with ASD were constructed. Second, drawing upon the core symptom characteristics of children with ASD, we innovatively designed a structured, functional, and serialized video animation program comprising 12 sets, as detailed in **Table 3**. A video animation intervention training program based on this was subsequently formulated. Finally, the feasibility of this program was verified through scientific evaluation.

The participants were randomly assigned to one of two groups. The intervention group (n=19) received intervention materials tailored to the novel art therapy model developed in this study, specifically 12 sets of innovatively designed video animations. The control group (n=19) received conventional video animation intervention materials. Both groups viewed videos with identical thematic content. The intervention cycle spanned 12 weeks, with one 45-minute session conducted weekly.

Compared to the control group, the intervention for the intervention group was deeply integrated with the PRT method on the basis of the same content. Each intervention session ensured a consistent PRT structure. First, for the target behavior and material design intervention, four scaled-down physical models were constructed. Video animations with corresponding difficulty levels, clear steps, well-defined goals, and progressive sequences were designed on the basis of the participants’ current ability levels. This ensured that the skills training helped improve their daily life skills (details in **Table 1**). Second, the perceptual preference adaptation intervention considered the color preferences, interaction preferences, and music preferences of children with ASD to ensure the smooth implementation of the intervention process and child engagement.

### 3.6 Procedure

#### 3.6.1 Randomization and blinding

The randomization sequence was generated by an independent research assistant who was not directly involved in intervention implementation, using a computer-generated random number table, with a 1:1 allocation ratio assigning 38 participants to the intervention group (n=19) and control group (n=19). The allocation sequence was sealed in sequentially numbered, opaque envelopes. After baseline data collection was completed and participants were confirmed to meet inclusion criteria, the same research assistant opened the envelopes sequentially and informed the instructors of the group assignments, thereby achieving allocation concealment.

Regarding blinding: due to the inherent differences between the two intervention conditions in material format (interactive video vs. standard video) and instructional procedures, instructors and parents could not be blinded to group allocation, which is an inherent characteristic of an open-label design. The two ATEC assessors did not access the random allocation information, did not participate in intervention delivery, and remained blinded to participant group assignment. Additionally, video recordings used for behavioral coding were subjected to information masking prior to coding, with file names retaining only participant ID and time identifier (pre-test/post-test). The two coders were blinded to research hypotheses, participant grouping, and time sequence. After coding completion, data were handed to a third-party statistician for pooled analysis, ensuring the objectivity and blinding of behavioral observation data.

#### 3.6.2 Preparticipation assessment

All the participants underwent a preliminary assessment to ensure that they met the research criteria. The screening criteria for participants included (a) being able to watch video animations and (b) being able to complete basic intervention activities under the guidance of a therapist.

#### 3.6.3 Reinforcer selection

Reinforcement is a key condition for shaping and maintaining target behaviors (48). This study used preference assessment to determine appropriate reinforcers for each individual. Through questionnaires and interviews with therapists, parents, and children, potential effective reinforcers aimed at enhancing motivation and intervention effects in video animation teaching were identified. The assessment revealed that the items preferred by child participants commonly included car toys, blind boxes, dinosaur eggs, and balloons. These items were used as incentives in subsequent interventions.

#### 3.6.4 Video animation intervention process

The interventions were conducted in a quiet, distraction-free classroom once per week for 12 weeks. Each session lasted 45 minutes, with the specific process as follows:

1. Task Explanation (approximately 5 minutes): The therapist introduced the task and basic requirements of the session.
2. Video Viewing and Interactive Operation (approximately 20 minutes): According to the intervention arrangement, the video animation was played. The therapist assisted the child in completing interface interactive operations but did not provide content prompts.
3. Generalization Training and Feedback (approximately 20 minutes): The child engaged in art activities or theme practices related to the course content. The therapist requested and recorded necessary behavioral feedback.

The control group used conventional video animations with the same themes as those used for the intervention group. The intervention process and duration were kept consistent. The entire intervention process was video recorded to document and analyze the behavioral performance of the children.

#### 3.6.5 Intervention design framework based on PRT

**Figure 3:**
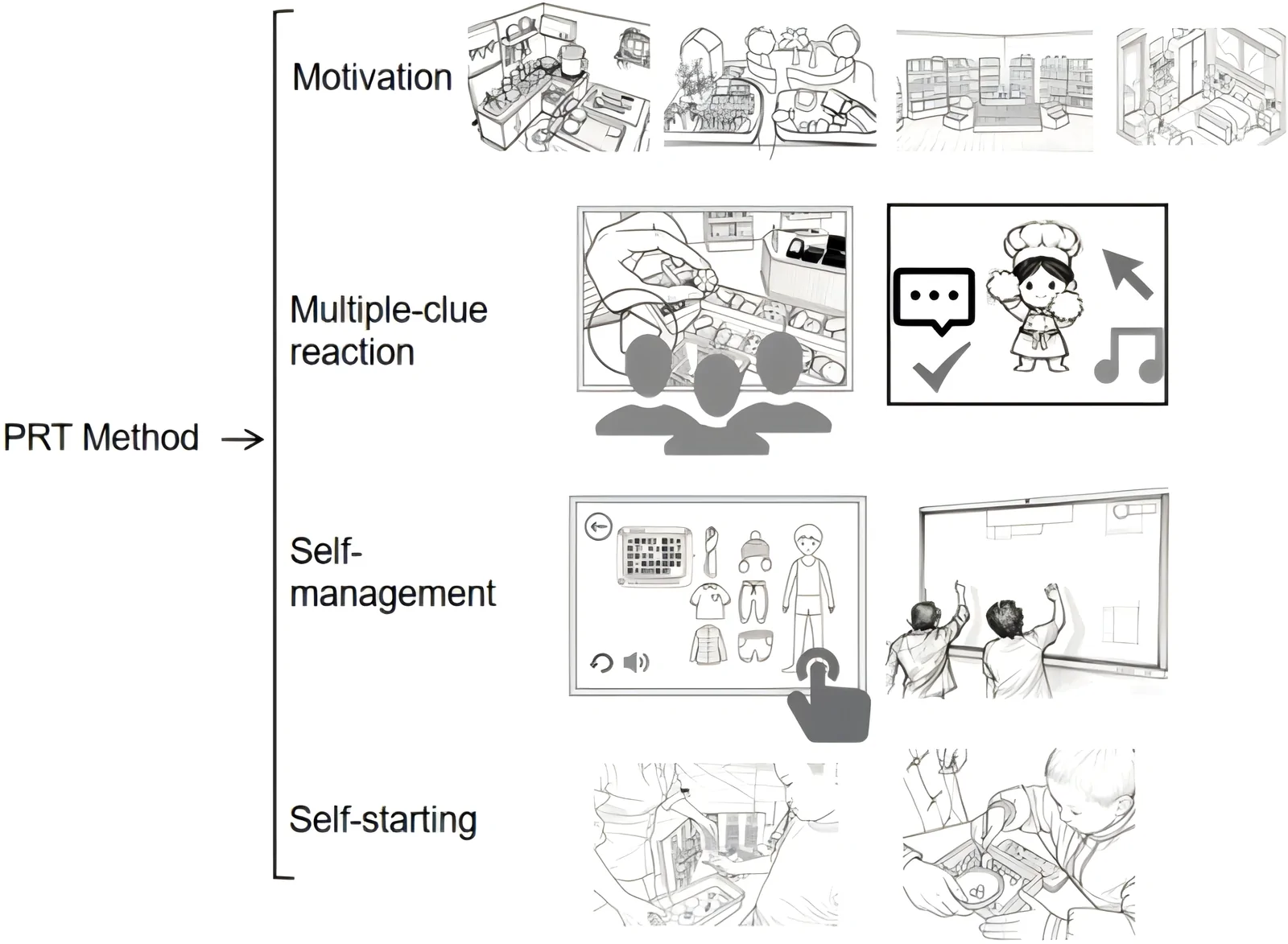
Intervention design framework for video animation incorporating PRT.

Combined with the theoretical model of PRT, a basic framework for intervention design and video animation content was constructed. First, in terms of intervention design, the structure of the PRT method was decomposed and mapped onto each module of the video animation content. Second, on the basis of the intervention goals, the video animations presented more specific content displays in terms of vision, sound, interaction (see **Figure 3**).

**Figure 4:**
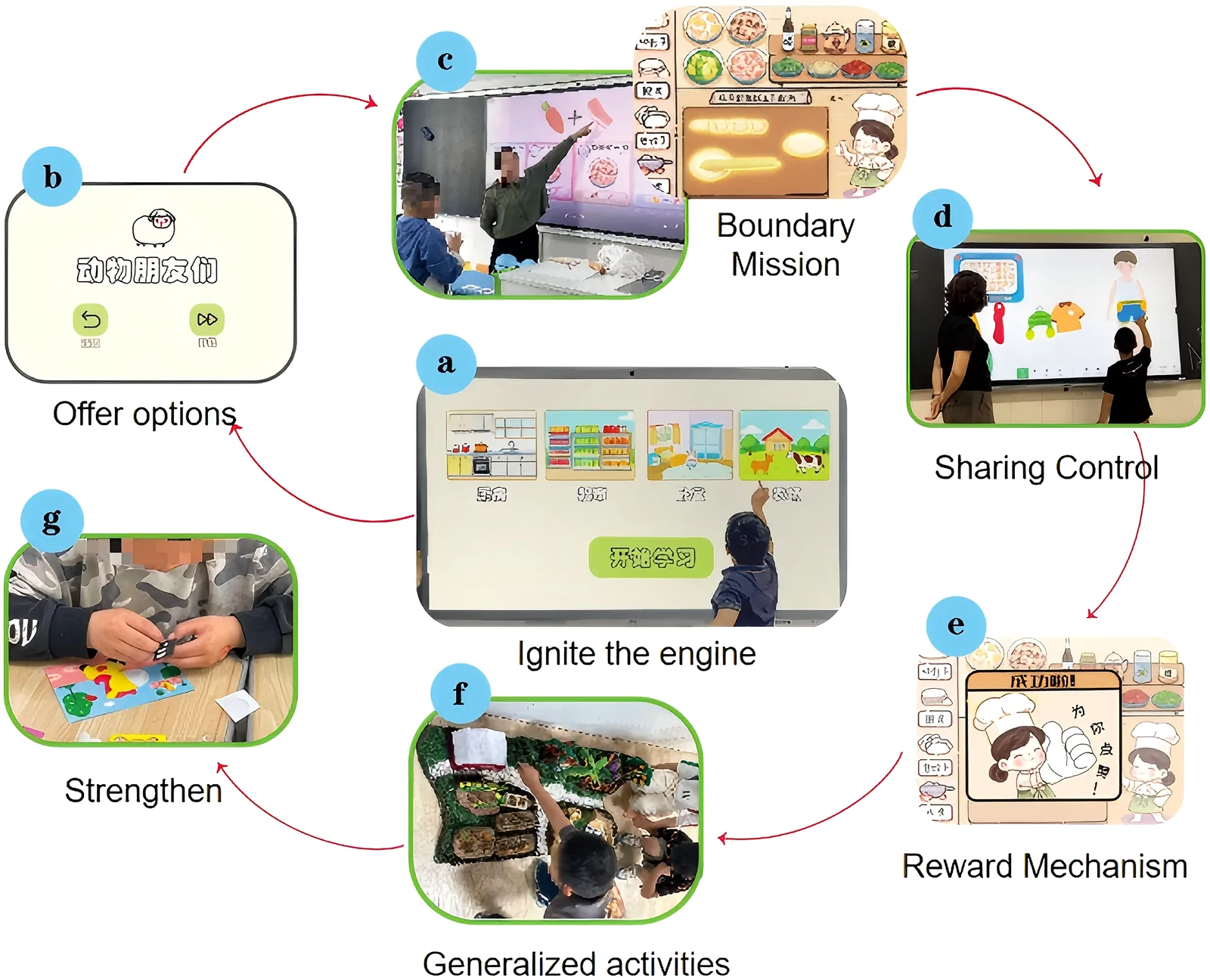
Process of video animation incorporating PRT. Each phase of the video animation corresponds to a specific PRT component: Phase 1 (Scenario Selection) → Motivating + Providing Choice; Phase 2 (Task Presentation) → Task Analysis; Phase 3 (Interactive Watching) → Sharing Control; Phase 4 (Feedback) → Reward Mechanism; Phase 5 (Practice) → Generalization Activities; Phase 6 (Reward) → Reinforcement.

**Figure 5:**
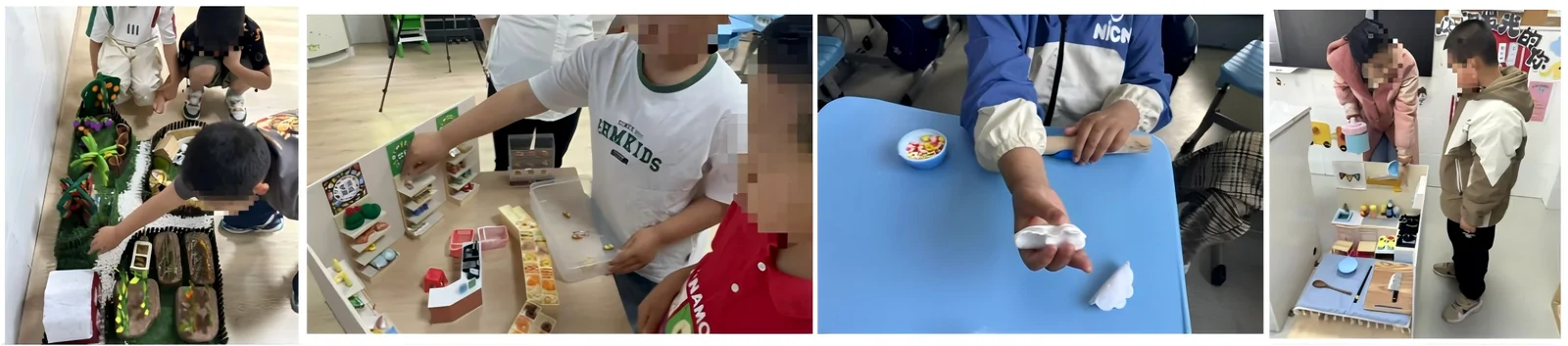
Intervention process diagram for children with ASD.

Specific Process: (a) Motivating: Following children’s interests, motivating participation through interactive interfaces with “scenario project” buttons. (b) Providing Choice: Respecting children’s choices, the interface provides content with different options for children to choose from. (c) Task Analysis: Providing clear tasks and opportunities, entering the video watching and learning phase, while the interface displays corresponding tasks. (d) Sharing Control: Children participate in interactive activities during video watching, thereby achieving active learning. (e) Reward Mechanism: Providing correct or incorrect response feedback through character or teacher feedback, stickers, etc. (f) Generalization Activities: Children receive immediate practice in generalization training. This includes not only interesting art activities but also scenario simulations. The scenario models are 1:1 recreations of real-life layouts, item placements, etc. This allows children to perform simulated practical actions in an environment consistent with the animation scene, transforming the processes understood from the video animation into concrete actions. (g) Reinforcement: Reinforcing the entire process by rewarding with a variety of toys, etc (see **Figures 3**–**5**).

#### 3.6.6 Generalization training

The generalization training in this intervention model follows the principles of “natural integration and progressive advancement”. Its key advantage lies in leveraging teaching opportunities from daily life, enabling children to consolidate learned skills in familiar, relaxed environments without adding extra training burden. The training process involved a progressive guidance strategy of “full assistance → partial prompting → independent completion,” supplemented by timely positive encouragement (e.g., verbal praise, small rewards). This gradually reduced children’s dependence on specific environments or objects, promoting initiative and flexibility in the application of skills.

To address the potential difficulty children with ASD experience regarding skill transfer (39), this design emphasizes the goal of “skill transfer, scenario application”. Ensuring that the training scenarios and materials are as close to real life as possible can help children effectively apply the skills learned in the classroom to practical situations. For example, training fine motor skills through the “tearing paper noodles” activity in class can naturally be generalized to simple household chores such as sorting vegetables or tearing vegetable leaves at home or creatively arranging food on a plate. This directly links skill practices with real-life needs.

Art activities, as the core component of this generalization training approach and a necessary part of art therapy, were designed to reflect the following three-dimensional advantages:

1. High Adaptability: The activity content is highly consistent with course themes and ability development goals, ensuring that the art form effectively serves educational intervention purposes.
2. Multi-Sensory Engagement: Integrating various hands-on and perceptual forms such as tearing, coloring, molding, and pinching accommodates the visual and practical learning characteristics of children with ASD while simultaneously promoting the development of multidomain abilities, including fine motor skills, cognitive understanding, and social cooperation.
3. Life Scenario-based: All activity designs originate from familiar daily scenes such as the kitchen, supermarket, and farm, reducing the cognitive load and cultivating children’s ability to learn from and adapt to life.

**Figure 6:**
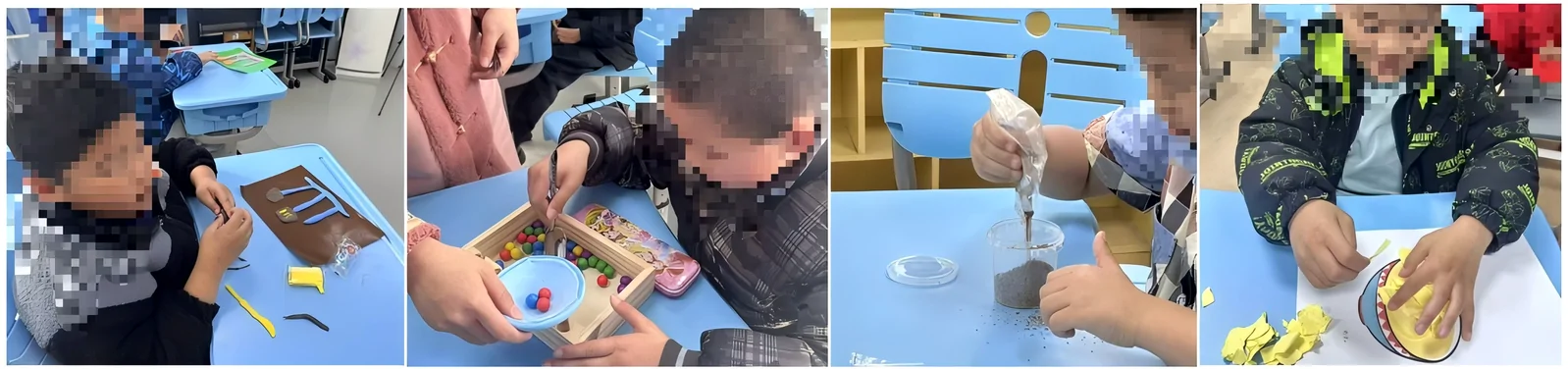
Activity process for children with ASD.

Furthermore, the operational materials (e.g., air-dry clay and coloring tools) were chosen to ensure safety, age appropriateness, and engagement, as they helped maintain the children’s motivation to participate in the intervention and enhanced the appeal and effectiveness of the intervention process. A schematic diagram of the generalization training design is shown in **Figure 6**.

### 3.7 Data collection

This study systematically collected data through three methods:

- Video Recording. A video camera recorded the entire process of pre−test, intervention, and post−test sessions. These recordings served as the basis for later quantitative analysis, qualitative evaluation, and intervention fidelity checks.
- Image Capture. A camera captured key behavioral moments of participants from multiple angles during the experiment, supplementing the video recordings.
- Questionnaires and Semi−structured Interviews. These were used to collect qualitative data on children’s participation and behavioral changes from parents and therapists, providing triangulation evidence for data analysis.

Social validity data were collected through the same interviews and a self−designed questionnaire. Interviewees included parents of all participating children (n=38) and their classroom therapists (n=4); each interview lasted 10–20 minutes and covered intervention acceptability, perceived usefulness, and observed changes in children’s daily behaviors. The questionnaire employed a 5−point Likert scale across four dimensions: enjoyment, perceived effectiveness, acceptability, and willingness to recommend. Interview transcripts were analyzed using thematic analysis to identify core themes.

### 3.8 Data analysis

All analyses were conducted on a per-protocol basis, as no participants withdrew or were excluded after randomization. SPSS 27.0 software was used for data analysis. Descriptive statistics were calculated for ATEC scores across the four dimensions pre- and postintervention. A 2 (group: intervention vs. control) × 2 (time: pre vs. post) repeated-measures ANOVA was conducted for each ATEC dimension to examine the intervention effects. Significant interaction effects were further explored using Bonferroni-corrected pairwise comparisons. Descriptive statistics for ATEC scores at pre- and post-intervention are presented in **Tables 4**, **5**.

**Table 4:** Descriptive statistical results of ATEC dimension scores before and after intervention for each group.

Dimension	Group	Number	Pre-test		Post-test	
			Mean (M)	Standard deviation (SD)	Mean (M)	Standard deviation (SD)
Language Communication	Int. Grp.	19	22.33	1.30	17.17	2.17
	Ctrl. Grp.	19	23.00	1.28	21.08	1.78
Social skills	Int. Grp.	19	27.25	0.87	22.08	1.62
	Ctrl. Grp.	19	27.33	0.78	25.08	1.31
Sensory/Cognitive Awareness	Int. Grp.	19	27.75	0.62	23.08	1.31
	Ctrl. Grp.	19	27.58	1.00	25.92	1.38
Behavior	Int. Grp.	19	8.50	0.52	5.92	0.79
	Ctrl. Grp.	19	8.75	0.62	7.42	0.51

SD, standard deviation. ATEC scores range from 02 for the first three dimensions (lower scores indicate better functioning) and from 03 for the behavior dimension.

**Table 5:** Mean change scores (post-pre) with 95% confidence intervals for both groups.

ATEC dimension	Intervention group change (M, 95% CI)	Control group change (M, 95% CI)	Mann-Whitney U	P	Cohen’s d
Language Communication	5.17 (4.59,5.75)	1.92 (1.34,2.50)	U=18.50	<.001	2.14
Social Skills	5.17 (4.39,5.95)	2.25 (1.73,2.77)	U=12.00	<.001	2.39
Sensory/Cognitive Awareness	4.67 (4.08,5.26)	1.67 (1.08,2.26)	U=30.00	<.001	1.94
Health/Physical Behavior	2.58 (2.28,2.88)	1.33 (1.03,1.63)	U=38.00	<.001	1.60

Change scores represent reduction in ATEC scores (post-pre, with negative change indicating improvement). All change scores are positive values indicating symptom reduction. CI, confidence interval.

A 2 (group) × 2 (time) repeated-measures ANOVA revealed significant time × group interaction effects for all four ATEC dimensions, indicating that the participants in the intervention group improved more than those in the control group did over the 12-week period. Mean change scores with 95% confidence intervals for both groups are shown in **Table 4**.

Language Communication: The interaction effect was significant, F(1, 36) = 67.74, p <.001, ηp² = .755. Simple effects analysis revealed that both groups improved significantly from pre- to postintervention (intervention: p*<.001, mean difference = 5.17, 95% CI [4.59, 5.75]; control: p <.001, mean difference = 1.92, 95% CI [1.34, 2.50]), but the magnitude of improvement was substantially greater in the intervention group.

Social Skills: A significant interaction effect was found, F(1, 36) = 80.69, p <.001, ηp² = .786. *Post hoc* comparisons indicated that the intervention group’s posttest scores (M = 22.08) were significantly lower than the control group’s posttest scores (M = 25.08), p <.001, mean difference = -3.00, 95% CI [-4.25, -1.75].

Sensory/Cognitive Awareness: The interaction was significant, F(1, 36) = 55.69, p<.001, ηp² = .717. The intervention group showed a mean reduction of 4.67 points (95% CI [4.08, 5.26]) compared with 1.67 points (95% CI [1.08, 2.26]) in the control group.

Health/Physical Behavior: The interaction effect was significant, F(1, 36) = 36.94, p <.001, ηp² = .627. The score of the intervention group improved by 2.58 points (95% CI [2.28, 2.88]), whereas that of the control group improved by 1.33 points (95% CI [1.03, 1.63]).

Baseline comparisons (pretest) revealed no significant differences between groups on any dimension (all p >.05), confirming successful randomization.

### 3.9 Intervention fidelity

To assess the accuracy of intervention implementation, two independent observers who were blind to group allocation randomly selected 10 classroom video recordings and evaluated them using a self-developed intervention fidelity checklist. The checklist was developed based on the intervention protocol and reviewed by three special education experts. The same fidelity criteria were applied to both conditions, with additional PRT-specific items added for the intervention group to ensure that PRT components were delivered as intended. The checklist assessed four aspects: (1) Intervention Content——prescribed intervention steps were executed without adding course-irrelevant content; (2) Intervention Duration——the intervention time strictly adhered to the 45-minute limit; (3) Implementation——clear and concise instructions were provided, with timely reinforcement; and (4) Environment——the setting was free from distractions, with all experiments conducted in grade-level classrooms that maintained consistent structural configurations. Scoring employed a 4-point Likert checklist (1 = not implemented, 2 = partially implemented, 3 = basic implementation, 4 = fully implemented). The fidelity coefficient was calculated as (total score ÷ maximum possible score) × 100%. Results indicated that both the intervention and control groups maintained high intervention fidelity, at 95.71% and 94.28%, respectively, demonstrating accurate and consistent implementation of the intervention protocols across both groups.

## 4 Results

Descriptive statistical results revealed that the posttest mean scores for the intervention group on all four dimensions decreased compared with the pretest scores, indicating a reduction in symptom severity and a trend toward functional improvement. The specific data are shown in **Figure 1**; **Table 1**. The results are organized to address the three research questions formulated in the Introduction。

### 4.1 Behavioral observation

The behavioral observation data directly address Research Question 2 by providing evidence on which specific design factors--particularly visual consistency and progressive task scaffolding--contributed to observable behavioral changes. The observed increases in verbal initiation and social interaction from early to late intervention stages suggest that the cross-media consistency between video animations and physical models reduced cognitive load and facilitated the transfer of learned behaviors to generalization contexts.

A self-developed behavioral observation checklist was used for recording. To assess the reliability of the observation results, two independent observers randomly coded 30% of the intervention videos and calculated the interrater agreement. The agreement coefficient was calculated as follows: (number of consistently recorded instances ÷ total recorded instances) × 100%. When this coefficient was greater than 90%, the observation results were considered reliable. The calculated agreement coefficients for each group at each stage were all above 95%, indicating that the behavioral data were reliable.

The observation results revealed that most of the children liked and accepted the teaching course and art activities. They were also interested in the teaching aids and content. A few children initially showed resistance but gradually cooperated with the teacher to complete tasks during the later stages of intervention. The changes in specific behavioral indicators before and after the intervention are shown in **Table 6**.

**Table 6:** Pre- and post-intervention behavioral changes within the intervention group (n=19).

Observation content	Subcategory	Intervention group (n=19)	Post-test	Test statistic (Z)	*p*-value
Verbal Initiation (Raising hand/Speaking)	No Participation	55% (10)	10% (2)		
	1–2 Times	30% (6)	55% (10)	-2.89	0.004
	> 2 Times	15% (3)	35% (7)		
Social Interaction	No Participation	55% (10)	10% (10)		
	1–2 Times	30% (6)	20% (4)	-3.62	<0.001
	> 2 Times	15% (3)	70% (13)		
Behavioral Performance (Classroom compliance)	No Relevant Behavior	35% (7)	10% (2)		
	1–2 Times	50% (9)	70% (13)	-2.41	0.016
	> 2 Times	15% (3)	20% (4)		

Values in parentheses indicate the number of children. Percentages are calculated as (number of children exhibiting the behavior at least once during observation ÷ total group size) × 100%. Pre-to-post comparisons were conducted using the Wilcoxon signed-rank test for ordinal categorical data (0, 1-2, >2 times per session). Z-values and p-values are based on the paired ordinal scores of the 19 children.

### 4.2 Social validity

Social validity data were collected through semi-structured interviews and a self-designed questionnaire. Interviewees included parents of all participating children (n=38) and their classroom therapists (n=4); each interview lasted 10–20 minutes and covered intervention acceptability, perceived usefulness, and observed changes in children’s daily behaviors. The questionnaire employed a 5-point Likert checklist across four dimensions: enjoyment, perceived effectiveness, acceptability, and willingness to recommend. Interview transcripts were analyzed using thematic analysis to identify core themes, with coding performed independently by two researchers and disagreements resolved through discussion. Three representative cases (A04, A06, A03) were selected for in-depth qualitative illustration based on their typicality of observed changes. To assess the social validity of the intervention, researchers conducted questionnaires and interviews with most parents and therapists after the intervention to understand their satisfaction with the intervention effects (49).

Parents and therapists generally held a positive attitude toward the intervention effects. They reported that the video animation design and extended art activities positively affected the children’s cognitive ability, social skills, and language expression. Some examples are as follows: The parent of participant A04 reported that the child previously often took food without asking. After the intervention, the child began to use social language, such as “Can I eat this?”.

Participant A06 was emotionally excitable and resistant at the beginning of the intervention. Later, the participant’s emotions gradually stabilized. The child was able to listen quietly and attempted to interact with therapists and peers. Participant A03 was originally verbally reserved and needed prompting to respond. After the intervention, the child was able to ask for help during generalization training. The child also initiated social dialog in related theme courses (e.g., mentioning “Having spicy hot pot with grandma at noon”).

### 4.3 Generalization outcomes

To provide evidence for cross-environment generalization (Research Question 3), we analyzed children’s performance during generalization training sessions that utilized 1:1 physical scene models. The percentage of children in the intervention group showing “ no observable behavioral response” decreased from 35% before the intervention to 10% after the intervention (see **Table 1**), indicating that this paradigm effectively stimulated participation motivation and behavioral expression in children. Notably, the physical model environment--including simulated kitchen, supermarket, bedroom, and farm setups--provided contextualized spaces where children could practice skills learned from the video animations. Behavioral observations during these sessions revealed that children in the intervention group were more likely to initiate learned behaviors (e.g., using play money correctly, sorting utensils appropriately) in the physical model context than in unstructured free-play settings.

However, it should be noted that this study did not calculate a quantitative “cross-media behavioral transfer rate” (i.e., the percentage of steps from the video animation correctly reproduced in the physical model). This limitation is addressed in the Discussion.

### 4.4 Individual changes in ATEC dimensions

To illustrate the pattern of individual responses to the intervention, **Figure 6** presents the pre- to post-intervention changes for each participant across the four ATEC dimensions. As shown, the majority of participants in the intervention group (15/19, 78.9%) demonstrated reduction exceeding the minimal clinically important difference across all four dimensions, whereas only 5/19 (26.3%) participants in the control group showed consistent improvements across all dimensions. This individual-level analysis complements the group-level findings by revealing substantial heterogeneity in treatment response, with some participants showing marked improvements while others showed minimal change.

## 5 Discussion

### 5.1 Dimension analysis

With respect to the language communication dimension, there was a significant difference in the changes in ability between the two groups of children before and after the intervention. The mean score reduction for the intervention group (M = 5.17, SD = 1.19) was significantly greater than that for the control group (M = 1.92, SD = 0.67), F(1, 36) = 67.74, *p* <.001, ηp² = .755, indicating a more pronounced improvement in language communication among the participants in the intervention group. These results suggest that the video animation intervention model incorporating PRT has greater advantages in terms of enhancing the language communication ability of children with ASD. This ability encompasses functions such as language comprehension and expression, nonverbal communication, communicative intent, and interactive language use. This advantage may stem from the use of scene models to replicate animation content within the intervention group, which helped to promote the transfer of knowledge and behaviors learned from the animations to real life. Individuals with autism often rely on images for learning; visual tools can effectively assist in their understanding of abstract rules (50). Therefore, in this study, video animations presented task processes visually, and scene models provided concrete visual support. This dual visual strategy fully utilized the visual processing strengths of children with ASD and avoided the issues ASD children face with auditory information in traditional language interventions, thereby enhancing the intervention efficacy. For example, in the Supermarket Chapter: Going Shopping, structured plots in sections such as “shopping” and “finding items” helped children understand processes and item functions. In generalization training, the 1:1 replicated supermarket scene model subsequently allowed children to perform simulated actions such as shopping and paying in a simulated environment consistent with the physical one, transforming what they learned from the animation into concrete actions. Consequently, the children were more willing to try, were more likely to participate, and were more likely to engage in language practices during the intervention process, resulting in more significant progress.

With respect to the social functioning dimension, the between-group difference also reached statistical significance. The mean score reduction for the intervention group (M = 5.17, SD = 0.83) was significantly greater than that for the control group (M = 2.25, SD = 0.75), F(1, 36) = 80.69, *p* <.001, ηp² = .786.

These findings indicate that the intervention group intervention had a more pronounced effect on enhancing the social functioning dimension. This dimension covers aspects such as social awareness, interaction ability, and social skills. These results are closely related to the intervention design: The video animations viewed by the intervention group contained numerous simulations of natural social situations, avoiding use of the mechanical, decontextualized training methods common in traditional interventions. This aligns with the core PRT principle of being “child-interest oriented, cultivating skills within authentic interactions” (51). Simultaneously, this paradigm also resonates with the perspective of social stories intervention theory. It posits that children with ASD process structured, visual social information more efficiently. Concrete situational presentations help them understand social rules and cues (52, 53). The use of video animations to deconstruct social processes through plots is a practical application of this theory. For example, in the Kitchen Chapter: Making Dumplings, sections such as “rolling dough” and “wrapping dumplings” teach the children how to engage in social behaviors such as passing tools, seeking help, and cooperating within tasks. The behavioral observation data also revealed that the percentage of children in the intervention group who “actively participated in group cooperation” increased from 15% to 70% in the later stages of the intervention (see **Table 1**). This finding indicates that they gradually mastered the use of basic social skills in actual interactions.

With respect to the sensory/cognitive awareness dimension, analysis of the between-group difference indicated that the magnitude of improvement for the intervention group (M = 4.67, SD = 1.15) was significantly greater than that for the control group (M = 1.67, SD = 0.78), F(1, 36) = 55.69, *p* <.001, ηp² = .717. These findings indicate that the intervention group’s intervention program focused more on improving the sensory processing abilities of children with ASD. This dimension covers manifestations such as sensory hypersensitivity, hyposensitivity, and seeking. Research has indicated that vision and hearing are sensory channels that can be effectively utilized in interventions (54). Therefore, the video animations for the intervention group provided children with rich perceptual input through multisensory stimulation and task analysis, which helped them gradually regulate their hypersensitivity or hyposensitivity to sensory information and enhance their ability to integrate information. Furthermore, improvements in sensory perception may be closely related to systematic generalization training. For example, in the Kitchen Chapter: Sweet, Sour, Bitter, Spicy, and Salty, taste perception was reinforced through activities such as “taste experience”. In the Farm Chapter series, activities such as observing planting and shaping animal features with clay formed a closed-loop “perception-action-cognition” training process. These findings echo those of Guanghui Li et al., who reported that cross-modal integration of tactile experiences with visual representations can significantly promote the enhancement of cognitive and narrative abilities (54).

With respect to the health/physical behavior dimension, the between-group difference was also significant. The mean score reduction for the intervention group (M = 2.58, SD = 0.51) was significantly greater than that for the control group (M = 1.33, SD = 0.49), F(1, 36) = 36.94, *p* <.001, ηp² = .627. These findings indicate that the PRT-based video animation intervention more effectively improved the daily behavioral norms and physiological functional adaptability of children with ASD. The video animations helped the children understand behavioral boundaries through concrete demonstrations of appropriate behaviors (e.g., safety demonstrations for “fires” and “knives” in the Kitchen Chapter: Recognizing Kitchenware). The “immediate feedback” mechanism embedded in the intervention (e.g., verbal encouragement from animated characters or therapists) further reinforced positive behaviors. The behavioral observation data also confirmed this finding. The percentage of children in the intervention group showing “ no observable behavioral response” significantly decreased from 35% to 10%.

Additionally, the intervention group intervention included an “immediate feedback” mechanism. By setting intuitive prompt symbols and rewards, each operation by the child received a precise, immediate response. This design not only aligns with the preference of children with ASD for predictable information but also reduces uncertainty in social interactions. For example, verbal praise and tangible rewards after children completed tasks reinforced their positive behaviors, promoting the generalization and active application of skills in real scenarios.

From the perspective of the overall intervention design, the contents of the 12 sets of course materials were structured in a progressive manner: the early sessions focused on foundational cognition, the midterm sessions emphasized social and language skills, and immediate generalization was integrated throughout the entire process. This structured and progressive design aligns with the learning characteristics of children with ASD and enables them to acquire new knowledge more effectively while consolidating preexisting skills, thereby demonstrating continuous progress.

Although the sample size was expanded to 38 participants, all were recruited from the same school district, which may limit generalizability. Future research could expand the sample size and include children from different regions to improve representativeness. To maintain the intervention schedule, completely individualized training based on individual differences was not implemented. Future studies could conduct long-term follow-ups with subgroups with different traits to explore the effects of personalized interventions.

The effect sizes reported in this study (ηp² ranging from.627 to.786) are relatively large for a small-sample intervention study. Several factors may account for this magnitude. First, the 12-week cumulative intervention produced sustained exposure to the treatment components. Second, the sample was relatively homogeneous (recruited from the same school and diagnostic source), which reduced within-group variance and consequently inflated partial eta squared values. Third, the intervention-control contrast involved a substantial difference in intervention intensity--the intervention group received a multi-component PRT package (video + physical models + generalization training + reinforcement), whereas the control group received linear video viewing only. Therefore, these effect sizes should be interpreted as representing the impact of the comprehensive PRT-based intervention package, rather than any single component. Our research question was not to isolate the independent effects of PRT or digital components, but to test whether the integrated PRT-informed digital intervention package could produce significant improvements across specific dimensions. Future replication studies with larger, more diverse samples are needed to obtain more stable effect size estimates.

### 5.2 Design factors contributing to intervention efficacy

**Figure 7:**
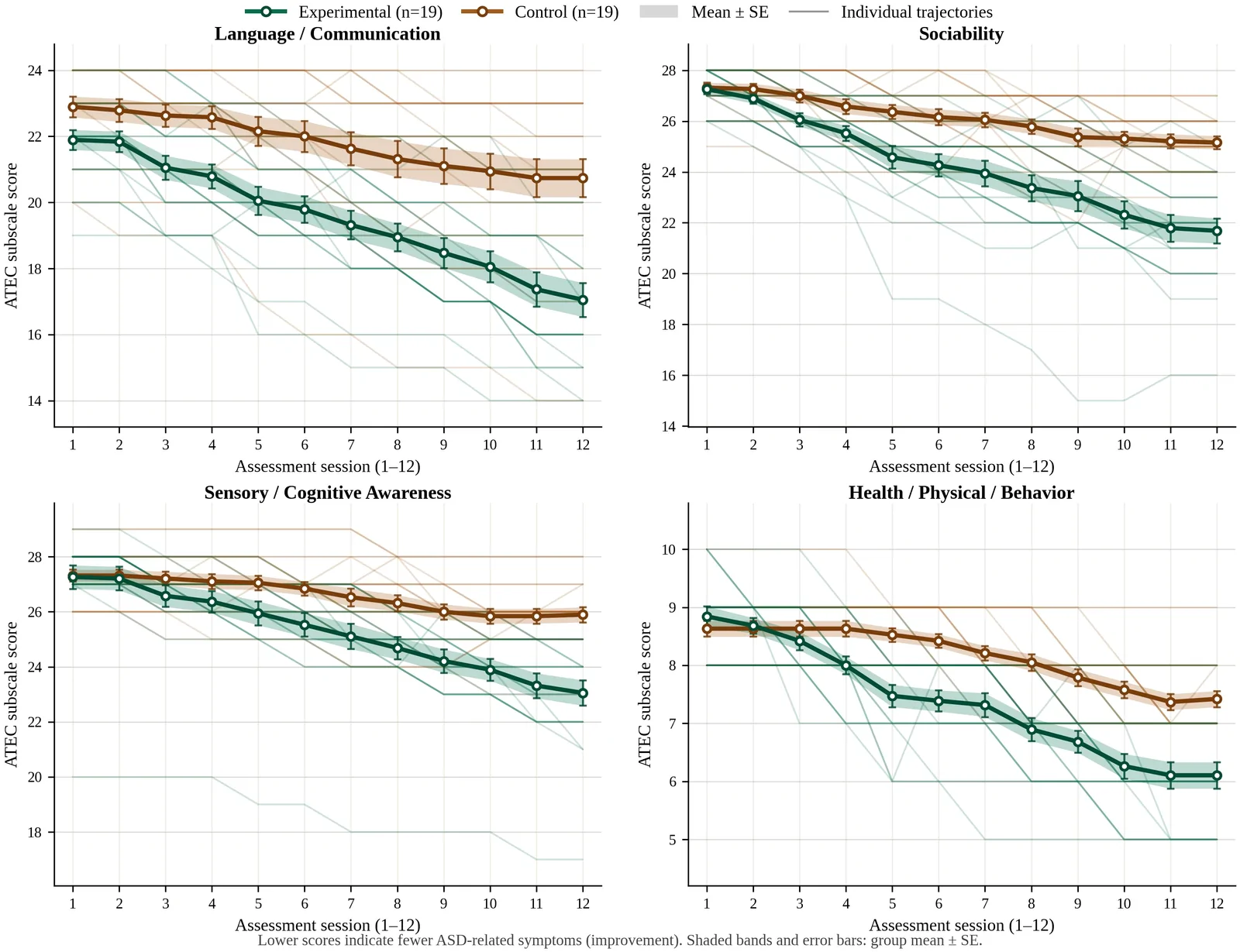
Individual change trajectories in ATEC dimensions. Blue lines: intervention group (n=19); gray lines: control group (n=19); bold lines: group means.

Beyond the overall effectiveness of the PRT-integrated model, this study identifies three design factors that appear critical to the observed improvements (55) (**Figure 7**):

1. Visual Consistency Across Media. The deliberate 1:1 replication between animation scenes and physical models reduced the cognitive distance between the “learning context” and “application context”. This design choice addresses a well-documented challenge in ASD interventions, the difficulty of transferring skills from training environments to real-life situations. Because of the minimization of contextual discontinuity, the children in the intervention group needed fewer prompts to initiate learned behaviors during generalization training.
2. Progressive Task Scaffolding. The 12-session curriculum was structured to progress from simple identification to complex social coordination. This scaffolding aligns with the “zone of proximal development” framework and allows children to accumulate successful experiences at each level before advancing. The significant between-group differences across all ATEC dimensions suggest that this structured progression—rather than isolated skill training—contributes to broad functional improvements.
3. Multimodal Reinforcement Integration. Unlike the reinforcement in the control condition, which relied primarily on verbal praise, the intervention group’s reinforcement was embedded across visual (animation feedback), social (therapist encouragement), and tangible (preferred items) channels. This multimodal approach accommodates the heterogeneous sensory preferences of children with ASD while maintaining the natural reinforcement principle central to PRT.

### 5.3 From design to mechanism: interpreting the psychological significance of design choices

The design elements identified above can be understood not only as technical specifications but also as psychological scaffolds that address the core processing characteristics of children with ASD. Specifically:

Cross-media visual consistency reduces the executive function burden associated with context switching, thereby freeing cognitive resources for use in social information processing. This interpretation is consistent with research suggesting that individuals with ASD rely greatly on visual processing.

Progressive task scaffolding mitigates the anxiety and avoidance behaviors often triggered by unpredictable or overly complex tasks, creating an environment with “predictable challenges” that sustains engagement while promoting skill acquisition.

Multimodal reinforcement accommodates the documented heterogeneity in sensory processing among children with ASD, ensuring that reinforcement is perceived as salient regardless of individual sensory profiles.

This analysis suggests a bidirectional relationship between design decisions and psychological mechanisms: design choices create the conditions under which therapeutic mechanisms can operate, and understanding these mechanisms provides a principled basis for refining future designs.

### 5.4 Practical implications for intervention design and implementation

The findings of this pilot study offer several practical implications for digital art therapy interventions for children with ASD. First, the paradigm provides a low-cost, high-fidelity approach deployable in areas lacking trained PRT therapists, enabling paraprofessionals or special education therapists to deliver structured interventions using standardized video animations and physical models. Second, the design framework offers standardized operational procedures that lower the implementation barrier for non-experts, as the structured 12-session curriculum combined with clear visual supports facilitates adequate fidelity. Third, the design principles identified—cross-media consistency, progressive task decomposition, and multimodal reinforcement—provide empirical evidence to guide the development of future digital art therapy applications for ASD populations. Finally, the detailed intervention protocol offers a replicable template adaptable to diverse cultural and educational settings.

### 5.5 Limitations and future directions

Several limitations of this study should be acknowledged. First, as a pilot investigation, the sample size is relatively small (38 participants, 19 per group), and all participants were recruited from the same school district with mild-to-moderate ASD, limiting external validity. Second, the absence of long-term follow-up assessments (e.g., at 3 or 6 months post-intervention) limits our ability to evaluate the durability of the observed treatment effects. While the 12-week intervention produced significant immediate improvements, it remains unclear whether these gains are maintained over time or whether booster sessions are needed. Due to project timeline and funding constraints, we were unable to conduct follow-up assessments in the current study. This is a critical gap that future research should address by incorporating systematic follow-up assessments. Third, as a multi-component intervention, the observed effects cannot be attributed to any single component (e.g., PRT alone, video animation alone, or physical models alone). The intervention group received a comprehensive package combining PRT-embedded video animations, 1:1 physical models, and structured generalization training, whereas the control group received only conventional linear video animations without PRT components. Therefore, it remains unclear whether the observed effects are driven primarily by the PRT principles, the digital medium, the cross-media consistency, or their interaction. Future research employing component analysis or dismantling designs (e.g., PRT + video vs. video alone, or PRT + physical models vs. PRT + video only) is needed to isolate the active ingredients of this multi-component intervention. Fourth, although the Mann-Whitney U test was appropriate for the non-normal distribution of change scores, future larger-sample studies may employ ANCOVA with baseline covariates or mixed-effects models to better control for regression to the mean. Fifth, the lack of a quantitative “cross-media behavioral transfer rate” limits direct evidence for the hypothesized generalization mechanism; future research should develop and validate such transfer metrics. Sixth, this trial was not preregistered in a clinical trial registry. While the intervention was behavioral and non-pharmacological, conducted in educational rehabilitation settings rather than clinical environments, preregistration remains an important practice for enhancing research transparency, reducing reporting bias, and specifying primary outcomes and analysis plans *a priori*. Future trials should be preregistered prior to participant enrollment. Seventh, the use of ATEC as the primary outcome measure—a comprehensive symptom checklist rather than a dedicated social functioning instrument—means that conclusions regarding social functioning improvement are indirect. Future studies should include specialized social functioning tools (e.g., SRS, VABS) for more precise validation.

## 6 Conclusion

This study structurally integrated the intervention logic of PRT into the entire digital art therapy media development process. The integration pathway is manifested primarily across three levels: The video animation design strictly adhered to the core steps of PRT (motivation, providing choice, task analysis, child initiation, immediate feedback, generalization training, and reinforcement). Twelve sets of thematic courses appropriate for improving the abilities of children with ASD were developed; these sets of courses extended the systematic intervention process from traditional settings to digital media. In constructing physical scene models, daily life scenes such as supermarkets and kitchens were replicated at a 1:1 scale, establishing a high degree of correspondence between the animated scenarios and real-world environments. In developing generalization training tools, accompanying physical operational materials with consistent themes (e.g., play money and tool cards) were designed to achieve visual and functional consistency between the digital interface and physical teaching aids. This integrated design helps children with ASD flexibly choose interaction methods during training and reduces interference from irrelevant stimuli.

The results indicate that the PRT-based digital art therapy program can effectively promote the development of social functioning in children with ASD across the four dimensions of speech/language communication, social functioning, sensory/cognitive awareness, and health/physical behavior. To synthesize the intervention effects, this study distilled key factors in the digital intervention design that help to enhance social functioning. First, a subgoal correspondence strategy was adopted in the content design. Clear, specific ability development goals were set around each intervention theme, ensuring that each course targeted specific social behaviors for intensive training. Second, visual consistency and narrative continuity were emphasized in the interface and character design. A unified protagonist and visual style were maintained throughout all the courses, providing the children with stable visual references and operational guidance, thereby reducing their cognitive load and adaptation pressure. Furthermore, multimodal interaction pathways were integrated into the generalization training module. Interactive digital games were combined with physical operational materials, leveraging the advantages of immediate feedback and dynamic demonstration of digital media while retaining the characteristics of tactile experience and real-world relevance of traditional teaching aids. This enhanced the children’s motivation to learn and sustained their participation.

The generalization training in this study followed the principles of “scenario consistency, ability progression, and natural extension”. Its realization is reflected across multiple levels: At the media level, a smooth transition from video observation to digital interaction and then to physical operation is achieved. At the scenario level, classroom tasks were organically extended to daily life situations. At the interpersonal level, children’s application of learned skills in real interactions was promoted by embedding cooperative tasks and elements such as “role-playing” and “group collaboration” into the tasks. The aforementioned designs significantly promoted attention, social, and language abilities in children with ASD, confirming the effectiveness and adaptability of using digital media for art therapy.

In the future, this paradigm can be developed to form a long-term art therapy intervention program. This will not only help further advance the precision and individualization of interventions for children with ASD but also provide practical references for the digital development of art therapy, thereby effectively broadening its application perspectives and development space within the field of special education.

In summary, as a pilot investigation, this study offers preliminary evidence and design insights for the emerging field of digital art therapy for children with ASD. First, it proposes a methodology for translating PRT principles into concrete design specifications for digital and physical therapeutic media. Second, it provides initial empirical evidence that cross-media design consistency-–maintaining visual and functional coherence between digital and physical training environments-–may enhance intervention outcomes across multiple functional domains. Third, it identifies specific design factors (visual consistency, progressive scaffolding, multimodal reinforcement) as potential mechanisms that warrant further investigation. Given the exploratory nature of this study, these findings should be interpreted cautiously, and replication with larger, more diverse samples is necessary before any claims of “validation” can be made. Future research should include 3- and 6-month post-intervention follow-up assessments to examine the long-term sustainability of treatment effects, employ component analysis or dismantling designs to isolate active ingredients, and incorporate specialized social functioning measures for more precise validation.

As digital technologies continue to reshape therapeutic landscapes, the systematic integration of theoretical models, user-centered design, and rigorous evaluation will be essential for developing interventions that are both effective and scalable.

## Data Availability

The original contributions presented in the study are included in the article/**Supplementary Material**. Further inquiries can be directed to the corresponding author.
