# Supplementary material for: A pilot randomized controlled study of a digital art therapy paradigm for children with ASD: integrating pivotal response treatment into video animation and physical model design
Source: Front Psychiatry. 2026 Aug 26;17:1894877. doi: 10.3389/fpsyt.2026.1894877 (PMC13555211; doi:10.3389/fpsyt.2026.1894877)
Supplement: Supplementary file 1 [file DataSheet1.pdf]

Anhui University of Technology
Ethics Review and Approval Form for Scientific Research Projects

**Application Date:**	October 15, 2024	**No.:**	YXLLSP20241003
**Project Title:**	Experimental Intervention with Video Animation for Children with Autism Spectrum Disorder		
**Project Principal Investigator:**	She Huili	**Mobile Phone:**	
**Affiliated School/Department:**	Anhui University of Technology/School of Art and Design	**Email:**	ysxyshl@ahut.edu.cn
**Collaborating Research Unit:**	Ma'anshan Special Education School	**Person in Charge:**	Du Juan
**Contact Number:**			
**Project Duration:** From __2024__ year __11__ month __1__ day to __2025__ year __7__ month __1__ day			
**Ethics Category:** ☐ **Biomedical** ☐ **Artificial Intelligence and Big Data** ☑**Humanities and Social Sciences** ☐ **Other**			
**Review Category:** ☑ **New Application** ☐ **Revised Project** ☐ **Continuing Review**			
**Project Research Content, Experimental Plan, List of Experimental Materials, etc.:**<br>Research into the Design of Digital Products for Art Therapy Interventions in Children with Special Needs Using Educational and Psychological Methods<br>This study explores the training efficacy and feasibility of these children in art therapy. It aims to establish an innovative model and intervention paradigm for digital research in art therapy for children with special needs.<br>All experimental materials employed during the process comprised artworks and art supplies developed by the research team, ensuring safety and harmlessness. By observing the reactions of children with special needs within these materials, insights into their cognitive, behavioural, and social characteristics can be gained, providing a basis for developing personalised intervention measures.			
**Commitment by the Applicant (Project Principal Investigator):**<br>All information provided above and all application materials are true and accurate. If approved, I will conduct the project strictly in accordance with the approved protocol and comply with the relevant regulations of the Anhui University of Technology Science and Technology Ethics Committee. If I violate the above statement, I am willing to bear the corresponding legal responsibility and all adverse consequences arising therefrom.<br>Applicant Signature:<br>Date:			
**Review Opinion of the University Science and Technology Ethics Committee:**<br>Chair of the Ethics Committee (Signature):<br>(Official Seal)<br>Date: October 15, 2024			

Remarks: This form is made in duplicate, one copy for the Ethics Committee and one copy for the applicant.
